# Elevated TATA-binding protein expression drives vascular endothelial growth factor expression in colon cancer

**DOI:** 10.18632/oncotarget.16384

**Published:** 2017-03-20

**Authors:** Sandra A.S. Johnson, Justin J. Lin, Christopher J. Walkey, Michael P. Leathers, Cristian Coarfa, Deborah L. Johnson

**Affiliations:** ^1^ Department of Molecular and Cell Biology, Baylor College of Medicine, Houston, Texas, United States of America; ^2^ Zymo Research, Irvine, California, United States of America; ^3^ Department of Orthopedic Surgery, University of California Los Angeles, David Geffen School of Medicine, Los Angeles, California, United States of America

**Keywords:** VEGFA, TATA-binding protein, colon cancer, gene expression

## Abstract

The TATA-binding protein (TBP) plays a central role in eukaryotic gene transcription. Given its key function in transcription initiation, TBP was initially thought to be an invariant protein. However, studies showed that TBP expression is upregulated by oncogenic signaling pathways. Furthermore, depending on the cell type, small increases in cellular TBP amounts can induce changes in cellular growth properties towards a transformed phenotype. Here we sought to identify the specific TBP-regulated gene targets that drive its ability to induce tumorigenesis. Using microarray analysis, our results reveal that increases in cellular TBP concentrations produce selective alterations in gene expression that include an enrichment for genes involved in angiogenesis. Accordingly, we find that TBP levels modulate VEGFA expression, the master regulator of angiogenesis. Increases in cellular TBP amounts induce VEGFA expression and secretion to enhance cell migration and tumor vascularization. TBP mediates changes in VEGFA transcription requiring its recruitment at a hypoxia-insensitive proximal TSS, revealing a mechanism for VEGF regulation under non-stress conditions. The results are clinically relevant as TBP expression is significantly increased in both colon adenocarcinomas as well as adenomas relative to normal tissue. Furthermore, TBP expression is positively correlated with VEGFA expression. Collectively, these studies support the idea that increases in TBP expression contribute to enhanced VEGFA transcription early in colorectal cancer development to drive tumorigenesis.

## INTRODUCTION

TATA-binding protein (TBP) is used for the transcription of genes from all three nuclear RNA polymerases (pols). TBP is recruited to promoters through either direct DNA binding, or indirectly through protein-protein interactions, to form active transcription initiation complexes. TBP expression is regulated transcriptionally through signaling pathways that involve protein kinase C, HBV protein X, EGFR1, and EGFRvIII via Ras [1–6]. These Ras-mediated events rely on activation of all three classes of mitogen-activated kinases [3, 5, 6]. Transcriptional regulation of TBP involves changes in transcription factor occupancy that converge on overlapping Elk1/AP1 binding sites within the TBP promoter [1, 3, 5–7]. The TBP promoter is also repressed through JNK2-dependent mechanisms and by direct recruitment of the repressor, Maf1, both of which inhibit Elk-1 occupancy [8, 9]. Thus, despite its central role in eukaryotic transcription, TBP expression is highly regulated through oncogenic signaling pathways.

Changes in cellular TBP expression affect the transcription of all three RNA pol-dependent processes. TBP can be limiting for both RNA pol I- and RNA pol III-dependent transcription which can then determine the cellular abundance of rRNAs and tRNAs [4, 9–11]. In contrast, changes in TBP expression have a selective and differential effect on RNA pol II-dependent targets and mRNA expression [3, 12–15]. Cellular changes in TBP have pronounced consequences on cellular growth properties depending on the cell type. Altering TBP expression affects cell proliferation rates of mouse embryo fibroblasts [6] and chicken DT cells [15], whereas enhanced TBP expression induces anchorage-independent growth and tumorigenesis in Rat1A cells [10, 16, 17]. Additionally, enhanced TBP expression is required for full oncogenic transformation by Ras [16]. While TBP-mediated transformation and tumorigenesis requires changes in RNA pol II-dependent transcription [16], the specific genes targeted to drive these phenotypic changes remain undefined.

VEGFA is a growth factor that is critical and rate limiting for angiogenesis [18–20]. VEGFA binds to target receptor tyrosine kinases to induce signaling events important to its functions in normal physiology, development, and pathological states where it is a central player, including tumor angiogenesis. VEGFA expression is regulated on multiple levels due to changes in transcription, mRNA processing and stability, and translation [21, 22]. Transcriptional regulation of VEGFA by oxygen tension and HIF-1 has been well studied. VEGFA transcription is induced via growth factors, cytokines, hormones, and oncogenes. This can occur independently of HIF-1, or serve to enhance hypoxia-mediated VEGFA induction [18, 20, 22]. The VEGFA promoter contains a transcription start site (TSS) at −1038 from the translation start site that has been widely shown to be hypoxia inducible [23, 24]. In addition, further analysis revealed the presence of an alternative TSS at −405 bp from the translation start site that is hypoxia insensitive [25].

Tumor development is limited without neovascularization [26] and inhibition of VEGFA impedes tumor growth [18–20, 27, 28]. Many tumor cell lines constitutively express and secrete VEGFA, indicating that deregulation of its expression is a common event in transformed cells. Furthermore, VEGFA is expressed by a vast majority of tumors and offers prognostic value for solid tumors [29, 30]. VEGFA expression is increased early in the pre-malignant state [27, 29–31]. Consequently, it is thought to play an important role early in tumor progression. However, relatively little is known about how VEGFA expression is regulated in the premalignant and early stages of tumor development when oncogenic mutations are acquired.

Our studies identify a new molecular node that connects growth factor signaling, TBP, and VEGFA expression to promote tumor development. Changes in cellular TBP amounts positively regulate VEGFA transcription and secretion. This is reflected in enhanced vascularization of cell line-based tumors that express increased TBP compared with control cells in tumorigenicity assays. TBP-mediated affects on VEGFA transcription occur through changes in the occupancy of TBP at the proximal promoter TSS, revealing an important function in regulating constitutive VEGFA expression for this previously poorly characterized transcription start site. Consistent with these findings, our analysis together with other *in silico* analyses reveal that TBP expression is elevated in a clinically significant number of human colon carcinomas relative to normal tissue. Enhanced TBP expression is also observed in colon adenomas supporting the idea that increased TBP expression may be an early event in colon cancer development. Furthermore, the relative TBP expression in colon tissues is positively correlated with VEGFA expression. Collectively, these studies support a new role for TBP in promoting tumor vascularization through its ability to modulate VEGFA expression.

## RESULTS

### Enhanced TBP expression produces select changes in RNA pol II-dependent genes

**Figure 1:**
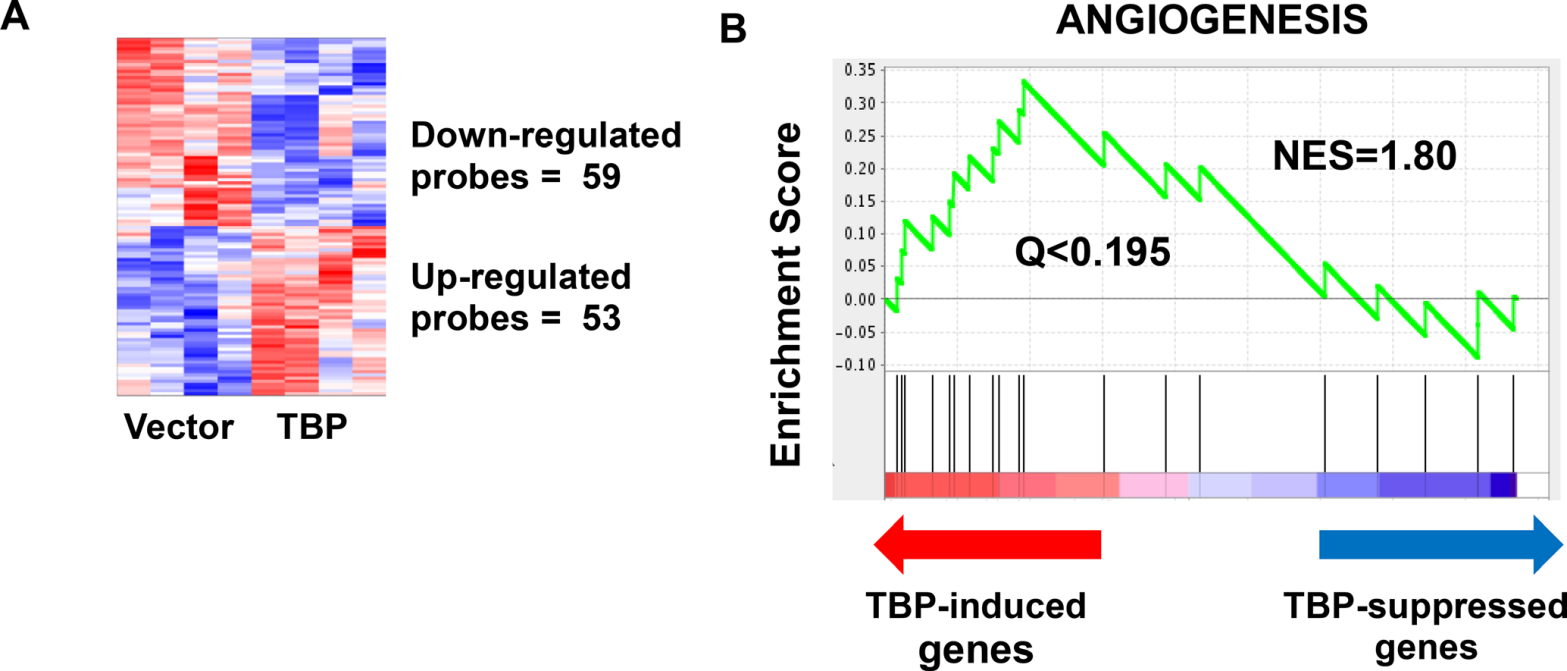
Microarray analysis reveals a modest but robust effect of TBP on global transcriptome (**A**) Increased expression of TBP in rat 1A cells resulted in 53 probes that are significantly increased (*p* < 0.05, fold change > 1.25) and 59 probes that are significantly decreased (*p* < 0.05, fold change < 0.8). (**B**) Gene Set Enrichment Analysis (GSEA) identifies an enrichment of the angiogenesis pathways (Normalized Enrichment Score/NES = 1.80, *Q* < 0.195).

While TBP is up-regulated by specific oncogenic signaling networks [1–3, 5, 6, 11] and enhanced TBP expression can transform cells and induce tumorigenesis [10, 16], the TBP-mediated changes in gene expression that facilitate these biological effects are not known. Previously, we showed that small increases in TBP protein expression (∼20%) in Rat1a (R1a) cells was sufficient to induce tumor formation in athymic mice [16]. However, R1a cells overexpressing mutant TBP proteins defective for RNA pol II-dependent transcription did not tumors [16] indicating that TBP-mediated tumorigenesis requires alterations in RNA pol II-dependent transcription. In order to identify TBP-mediated changes in gene expression that drive tumor formation, we conducted a microarray analysis to compare changes in gene expression in R1A cells that expressed increased amounts of TBP (R1a-hTBP) to vector control cells. These results revealed modest changes in gene expression by TBP with only 112 genes that displayed statistically significant changes of 1.25-fold or greater (Figure 1, Supplementary File 1). Of these changes, approximately half of the genes were upregulated and the other half was downregulated. Gene Set Enrichment Analysis (GSEA) further revealed that further revealed that TBP consistently alters the expression of genes involved in angiogenesis (Figure 1), (*Q* < 0.25, normalized enrichment score/NES = 1.80).

### Tumor formation mediated by increased TBP expression results in enhanced tumor vascularization

**Figure 2:**
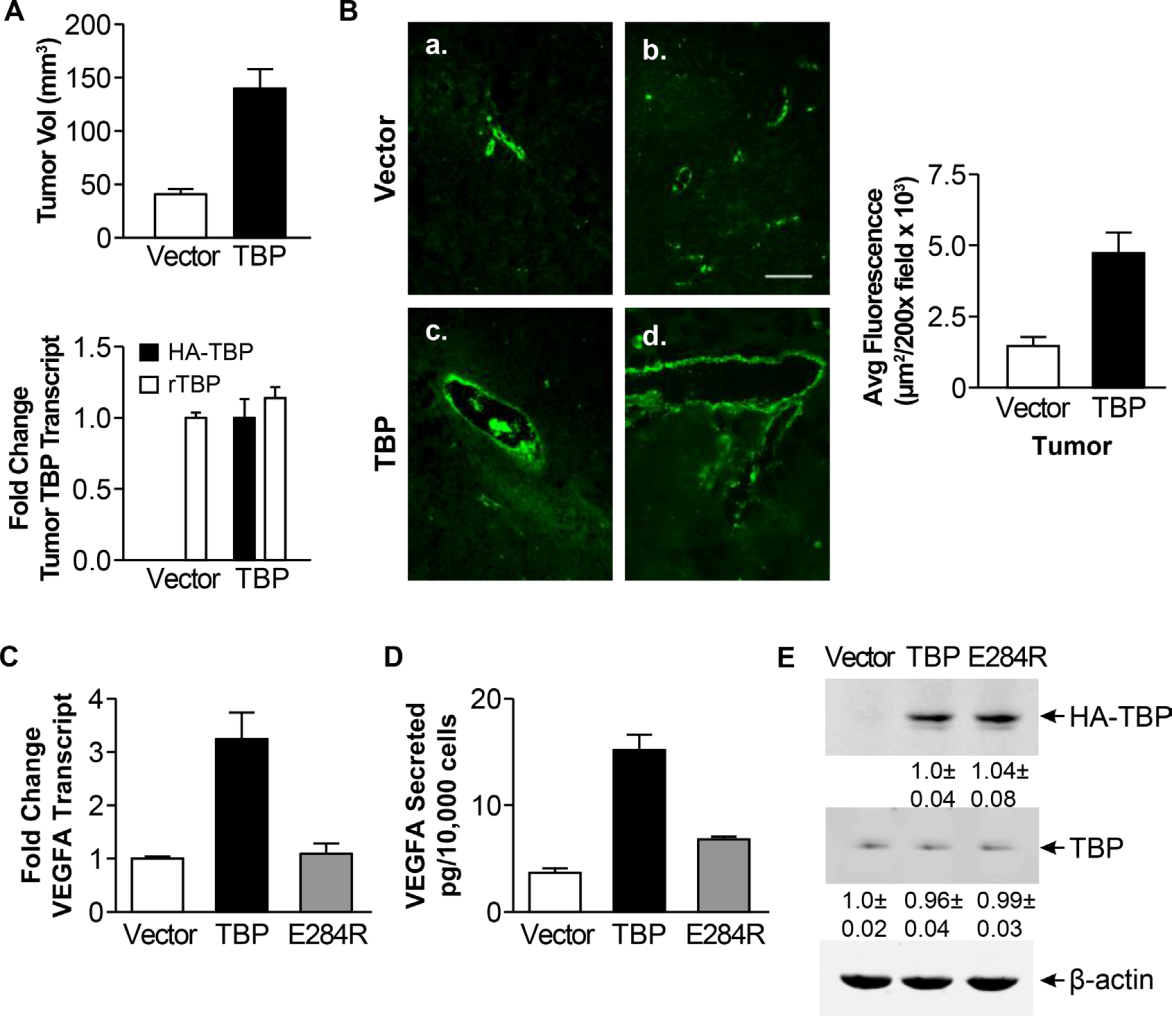
Rat1a cells with enhanced TBP expression form tumors with increased von Willebrand staining and exhibit increased VEGFA expression (**A**) Rat1A cells expressing HA-tagged human wild type TBP or empty expression vector (Vector) were used in tumorigenicity assays with athymic mice. *Top panel*. Comparison of xenograft tumor volumes. Tumor volumes were determined by measuring the dimensions (H x W x D) 36 days post-injection. *Bottom panel*. Levels of endogenous rat (r)TBP and ectopic HA-tagged TBP mRNAs in tumors derived from Rat1A stable cells determined by RT-qPCR. Fold changes are calculated based on GAPDH-normalized levels in tumors derived from vector cell line (rTBP) or set to 1 for ectopic TBP (HA-tagged hTBP). Values are means ± S.E. (*n* > 3, *p* < 0.0001). (**B**) vWF staining in tumor tissues derived from rat1a vector (a., b.) or TBP stable (c., d.) cells from “A”. Representative images from 3–5 independent tumors. *Right*. Quantification of vWF immunofluorescence. Average fluorescence was determined in μm^2^/200X field (> 25 fields per tissue section). Values (average ± S.E., *p <* 0.0001). Scale = 100 μ. (**C**) VEGFA transcripts analyzed by RT-qPCR from Rat1a stable cells wild-type or TBP-E284R. Transcripts were GAPDH-normalized. Fold changes based on transcripts in vector cells. Values (average ± S.E, *n* = 3). Statistical differences: TBP v. vector and E284R (*p* < 0.001). (**D**) VEGFA levels in conditioned media from Rat1a stable cells in “C” were determined by ELISA. Values (average ± S.E, *n* = 3). Statistical differences: TBP v. vector and E284R (*p* < 0.001). (**E**) Immunoblot analysis of Rat1a cells in “C”. Densitometry values were β-actin-normalized and compared to vector (endogenous TBP) or TBP cells (HA-TBP). Representative immunoblot shown.

Given the enrichment and consistent increase in TBP-mediated changes of expression for genes that regulate angiogenesis, we assessed whether enhanced TBP expression might support tumor growth by enhancing tumor vascularization. Tumors were examined from athymic mice injected with R1a cells stably overexpressing HA-tagged human TBP (Figure 2A). The tumor burden from these cells was found to develop sooner [10, 16] and consequently the tumors were larger than the tumors from the vector control cells (Figure 2A, *p* < 0.0001). Immunohistochemical staining of these tumors for von Willebrand factor (vWF), an endothelial cell marker, was used to visualize the tumor blood vessels (Figure 2B). Compared to the tumors derived from the control cells, the tumors from R1a-hTBP cells displayed increased vWF staining and qualitatively, the number and size of the blood vessels were also larger. This suggests that the R1a-hTBP cells possess an enhanced angiogenic program that supports blood vessel development early in tumor formation in this model system.

### Cellular TBP amounts regulate VEGFA expression and cell migration

Our microarray analysis revealed an enrichment of the angiogenesis pathway in the TBP transcriptomic footprint. VEGFA is a well-documented pro-angiogenic growth factor and its overexpression can permit cells with low levels of oncogenic Ras to become tumorigenic [28]. We therefore examined potential changes in VEGFA expression in the R1a-hTBP cells and in R1a cells expressing a TBP mutant defective in RNA pol II-dependent transcription, R1a-hTBP-E284R [32, 33]. Enhanced TBP expression produced increases in both VEGFA transcripts (Figure 2C, *p* < 0.001) and secreted VEGFA (Figure 2D, *p* < 0.001) compared to the control cells. In contrast, expression of TBP-E284R was unable to induce either increased VEGFA transcripts or increased secreted VEGFA, despite being expressed at relatively similar levels to the wild-type TBP (Figure 2E). Together, these results indicate that increased TBP, and altered RNA pol II-mediated transcription, induces expression and secretion of VEGFA in R1a cells.

**Figure 3:**
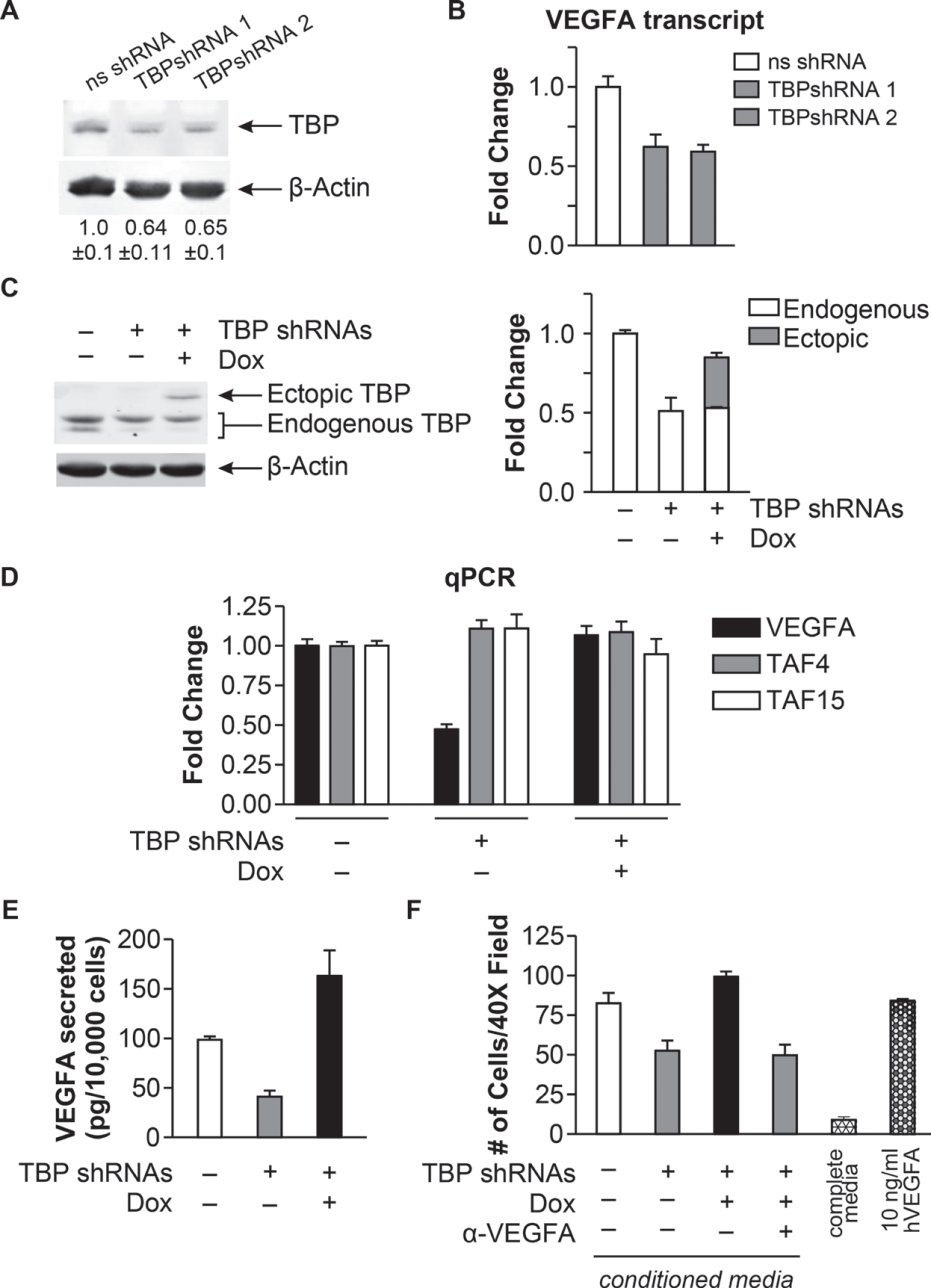
TBP concentrations regulate bioactive VEGFA expression and cell migration (**A**) Immunoblot analysis from HT-29 cells with ns- or TBP-shRNA. Densitometry values (average ± S.E.) are β-actin-normalized TBP with ns-shRNA value set to 1. (**B**) VEGFA transcripts determined by RT-qPCR from cells in “A”. Transcripts were GAPDH normalized. Fold changes based on levels in ns-shRNA control. Values (average ± S.E., *n* > 6). Statistical differences: ns-shRNA v. TBP-shRNA-1, TBP-shRNA-2 (*p* < 0.005). (**C**) Immunoblot analysis of HT-29 cells with inducible human HA-tagged TBP expression and ns- or TBP-shRNAs (1+2) treated with 30 ng/μl doxycycline (Dox). *Right*: Representative blot shown. *Left*: Densitometry of total TBP, endogenous + ectopic (HA-tagged) TBP. Fold change (average ± S.E.) of b-actin-normalized TBP compared to total TBP in ns-shRNA cells. (**D**) Transcripts determined by RT-qPCR from cells in “C”. Fold changes of normalized transcripts based on ns-shRNA levels. Values (average ± S.E., *n* > 6). Statistical differences: VEGFA, ns-shRNA and TBP-shRNA + Dox v. TBP-shRNA – Dox (*p* < 0.0001). (**E**) VEGFA levels in conditioned media from cells in “A” determined by ELISA and normalized to total number of cells. Statistically different values (average ± S.E, *n* > 5): ns-shRNA v. TBP-shRNAs – Dox (*p* = 0.04); ns-shRNA v. TBP-shRNAs + Dox (*p* = 0.02); TBP-shRNAs – Dox v. TBP-shRNAs + Dox (*p* < 0.0001). (**F**) Endothelial chemotaxis assay using conditioned media from cells in “A”. Values (average ± S.E., *n* > 5) represent the number of HUVECs migrating through transwell membranes (40X; 10 distinct fields per independent determination). a-VEGFA: conditioned media pre-treated with VEGFA antibody. Negative control: media with 10%FBS. Positive control: media with 10 ng/ml recombinant hVEGFA. Statistical differences: ns-shRNA v. TBP-shRNAs – Dox (*p* = 0.0071); TBP-shRNAs – Dox v. TBP-shRNAs + Dox; ns-shRNA and TBP-shRNAs + Dox v. TBP-shRNAs + Dox + a-VEGFA (*p* = 0.0002).

We next investigated whether changes in TBP expression could alter VEGFA expression in a transformed cell line using HT-29 human colon adenocarcinoma cells. TBP shRNA-mediated knockdown (Figure 3A) resulted in a significant decrease in VEGFA mRNA expression (Figure 3B, *p* < 0.005). Since the shRNAs target the 3′ UTR of TBP, we employed a doxycline-inducible system to restore TBP expression (Figure 3C). In this system, VEGFA mRNA levels were similar to the control levels when TBP was ectopically expressed together with endogenous TBP knockdown (Figure 3D). This is not a consequence of overall changes in global transcription as mRNAs for the TBP-associated factors, TAF4 and TAF15, were unchanged. TBP-induced changes in VEGFA resulted in similar changes in secreted VEGFA protein (Figure 3E).

To determine whether TBP-induced changes in VEGFA expression could stimulate endothelial cell migration, conditioned media from the HT-29 cells was used to measure the migration of HUVECs. Significantly less migration was observed when HUVECs were exposed to conditioned media from cells with reduced TBP compared to conditioned media from control cells, whereas re-expression of ectopic TBP was as potent as conditioned media from control cells in stimulating migration (Figure 3F). Furthermore, immunoblocking of the conditioned media with VEGFA antibody also inhibited HUVEC migration similarly to TBP knockdown, indicating that VEGFA is an important chemoattractant in the conditioned media. Together these results reveal that cellular TBP concentrations modulate the expression and secretion of bioactive VEGF.

### Altered TBP expression and its occupancy on the proximal VEGFA promoter drive VEGFA expression

**Figure 4:**
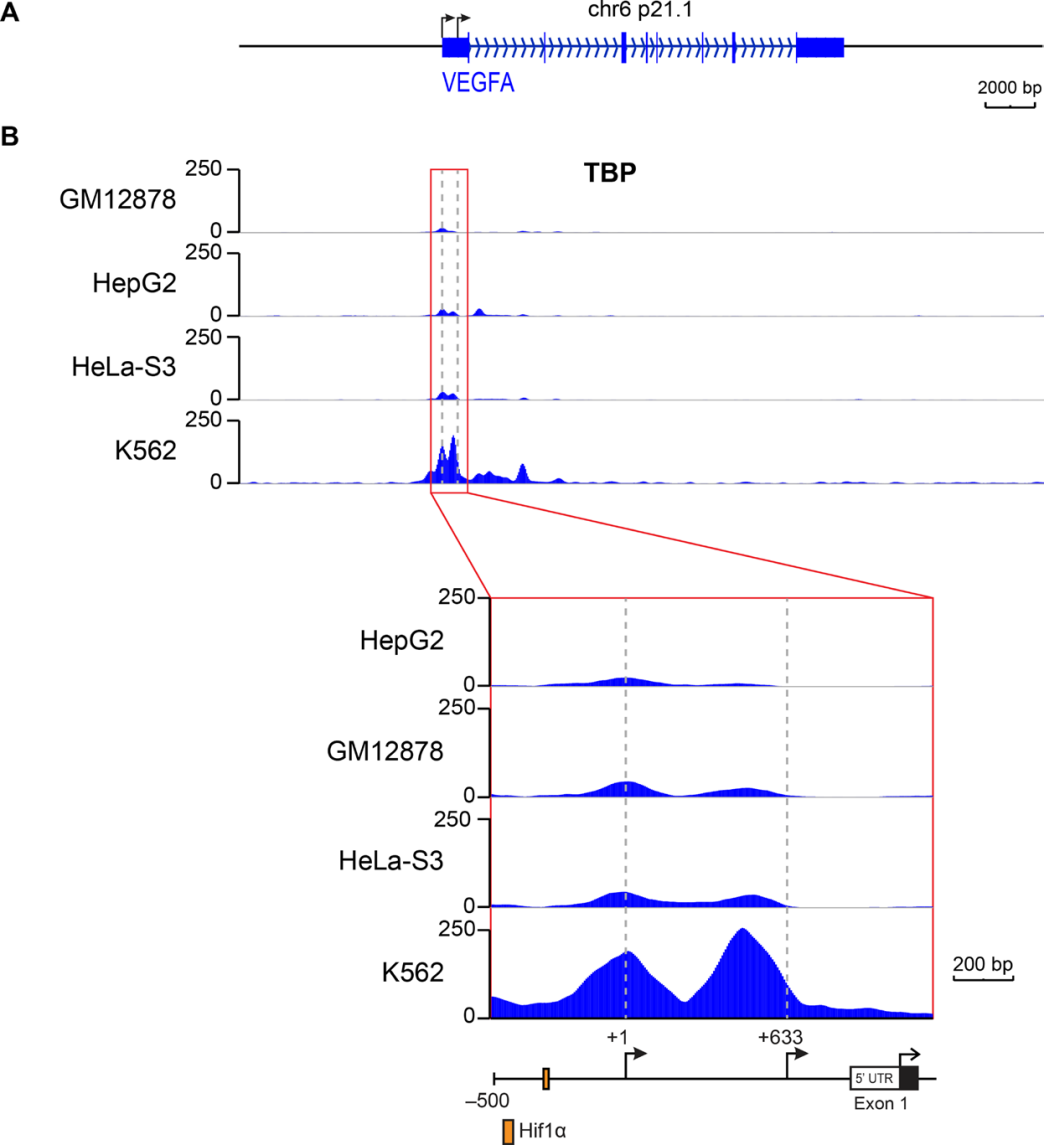
TBP is occupied at two distinct sites on the VEGFA promoter that encompass two transcription start sites TBP ChIP-seq data for the VEGFA gene in GM12878, K562, Hela-S3, and HepG2 cells from ENCODE. (**A**) Scaled promoter-gene schematic of VEGFA gene aligned with data tracks. (**B**) TBP binding for the VEGFA gene (*top*). Gray box highlights the region in *Inset* below. Inset shows binding in the promoter/5′ UTR region. Data tracks are .bigwig format. Dashed lines represent locations of distal (+1) and proximal (+633) TSSs.

To examine whether TBP was directly regulating VEGFA expression, we queried genome-wide chromation immunoprecipitation sequencing (ChIP-seq) data for TBP binding at the VEGFA promoter from published ENCODE data for four different cell lines expressing VEGFA transcripts under normal growth conditions [34]. Comparing TBP occupancy approximately 1000 bp upstream from the VEGFA mRNA translation start site to the average baseline TBP signal in the surrounding region ± 50 kb, we found that TBP was enriched approximately 10-15 fold in the VEGFA promoter region (Figure 4B). TBP occupancy was found at two distinct peaks within this region, one corresponding to the well studied distal TSS (+1, −1038 bp from the translation start site) and the other encompassing the non-conventional hypoxia-independent proximal TSS (+633, −408 bp from the translation start site, Figure 4A–4B).

**Figure 5:**
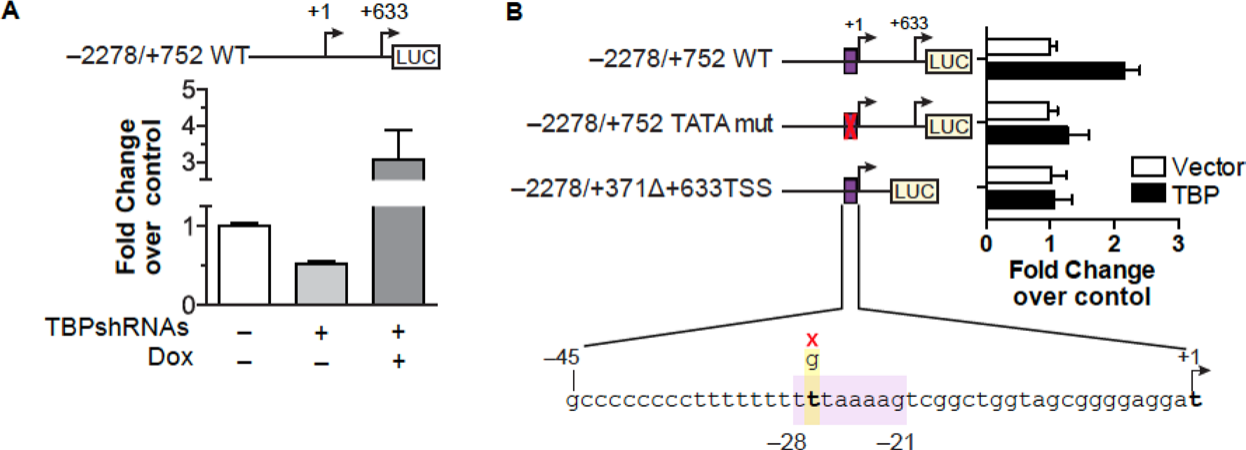
TBP-mediated induction of VEGFA expression requires sequences within both regions of the proximal and distal TSSs (**A**) HT-29 cells with inducible TBP ± TBP-shRNAs transfected with VEGFA promoter-luciferase construct containing both transcription start sites (*top*, schematic). Luciferase normalized to β-galactoside. Values (average ± S.E., *n* > 5) set to activity in ns-shRNA cells. Statistical differences: ns-shRNA v. TBP-shRNAs – dox (*p* = 0.04), ns-shRNA and TBP-shRNAs – dox v. TBP-shRNAs + dox (*p <* 0.0001). (**B**) Cells co-transfected with a control or TBP expression vector, CMV-β-galactoside vector, and human VEGFA promoter-luciferase construct (−2278/+752 WT, −2278/+752 TATA mut, or −2278/+371D+633TSS WT). Schematics (left) show TSSs and the TBP binding site for VEGFA promoter-reporter constructs. TATA mutagenesis shown below. Values (average ± S.E., *n* = 6) are based on normalized luciferase in control cells for each promoter-reporter. Statistical differences:–2278/+752 WT, Vector v. TBP (*p <* 0.0001).

To determine whether TBP-dependent changes in VEGFA mRNA expression were due to changes in VEGFA transcription, a VEGFA promoter-luciferase construct was examined. VEGFA promoter activity was negatively regulated by reductions in cellular TBP, while restoring TBP levels induced promoter activity (Figure 5A). A genome wide computational analysis of human core promoters previously identified a TATA box (HWHWWWWR) between −28 to −21 of the VEGFA promoter [35] (Figure 5B). This sequence was mutagenized to determine if direct TBP binding at this site would affect promoter activity. This mutation had no effect on the overall promoter activity but rendered the construct insensitive to increased TBP expression. Deletion of the downstream proximal TSS, which does not contain a consensus TATA sequence, also abrogated TBP-induced changes VEGFA promoter activity, indicating that upstream sequences that include the distal TSS are not sufficient to modulate TBP-mediated changes in VEGFA transcription. Thus, both the proximal and distal TSS regions are necessary for TBP-mediated regulation of VEGFA expression.

**Figure 6:**
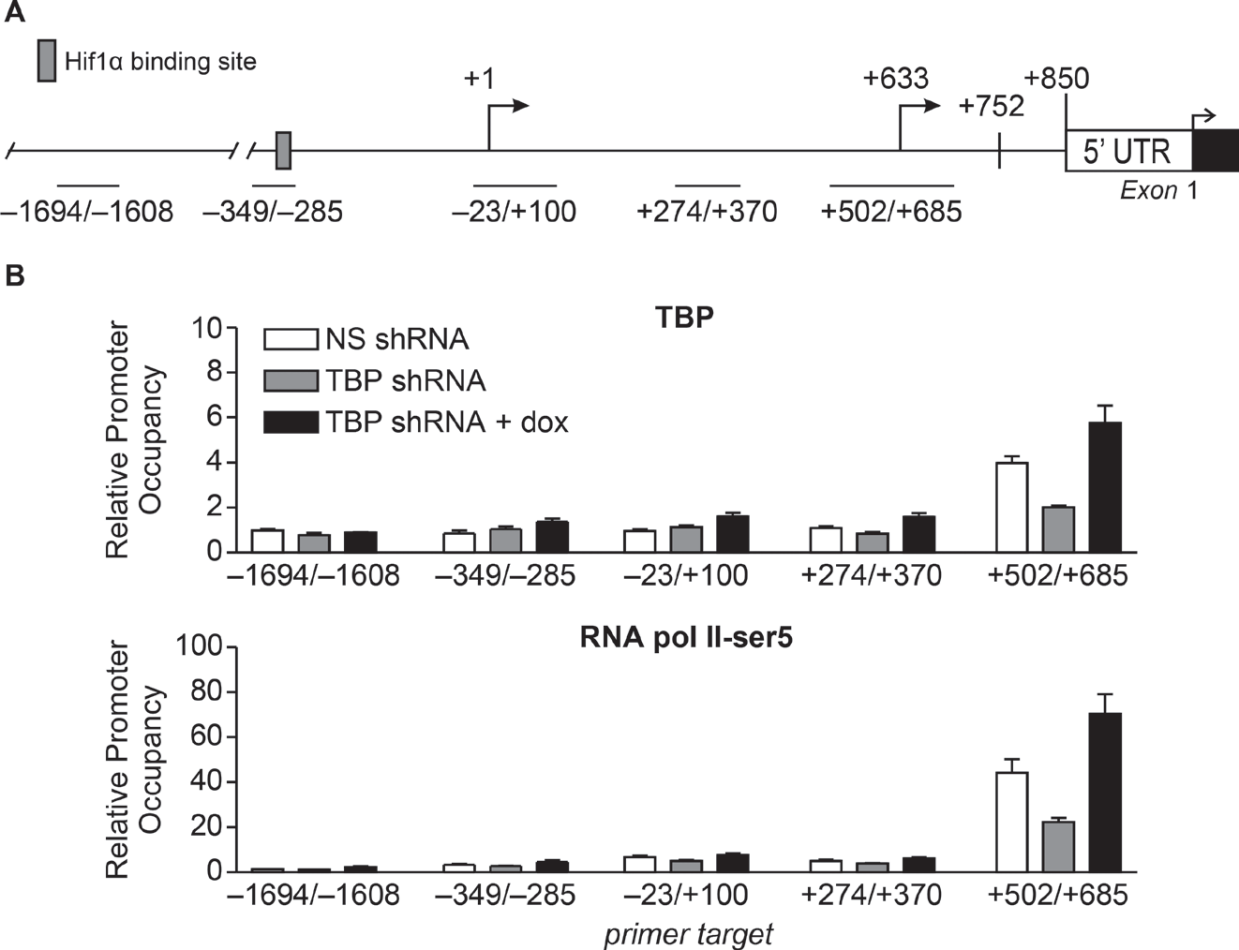
Alterations in TBP expression are positively correlated with occupancy of TBP and initiated RNA pol II at the proximal TSS (**A**) Schematic of VEGFA 5′ UTR including proximal (+633) and distal (+1) TSSs. Lines represent regions primed for qPCR-ChIP analysis. (**B**) ChIP analysis using HT-29 dox-inducible TBP ± TBP-shRNAs cells and TBP (*top*) or RNA polymerase II-Ser5 (*bottom*) antibodies. Relative promoter occupancy is normalized to 10% input and IgG pull down. Values (average ± S.E., *n* = 3). Statistical differences: TBP pull-down, +502/+685, ns-shRNA, and TBP-shRNAs + dox v. TBP-shRNAs (*p* < 0.0001); ns-shRNA v. TBP-shRNAs + dox (*p* = 0.0004); RNA pol II pull-down, +502/+685, ns-shRNA, and TBP-shRNAs + dox v. TBP-shRNAs (*p* < 0.0001); ns-shRNA v. TBP-shRNAs + dox (*p* < 0.0001).

ChIP analysis was used to detect TBP occupancy on the regions encompassing the VEGFA TSSs when cellular TBP levels were altered (Figure 6A). Only the region that included the proximal TSS displayed changes in TBP occupancy in response to altered TBP expression (Figure 6B). These changes further correlated with changes in initiated RNA polymerase II occupancy at the proximal TSS. Furthermore, no significant detection of TBP or RNA polymerase II was observed at the distal TSS relative to the proximal TSS. Collectively, our data support the idea that TBP-mediated regulation of VEGFA transcription occurs through changes in TBP expression and its occupancy on an alternative TSS *in vivo*.

### TBP is upregulated in human colon tumors

**Figure 7:**
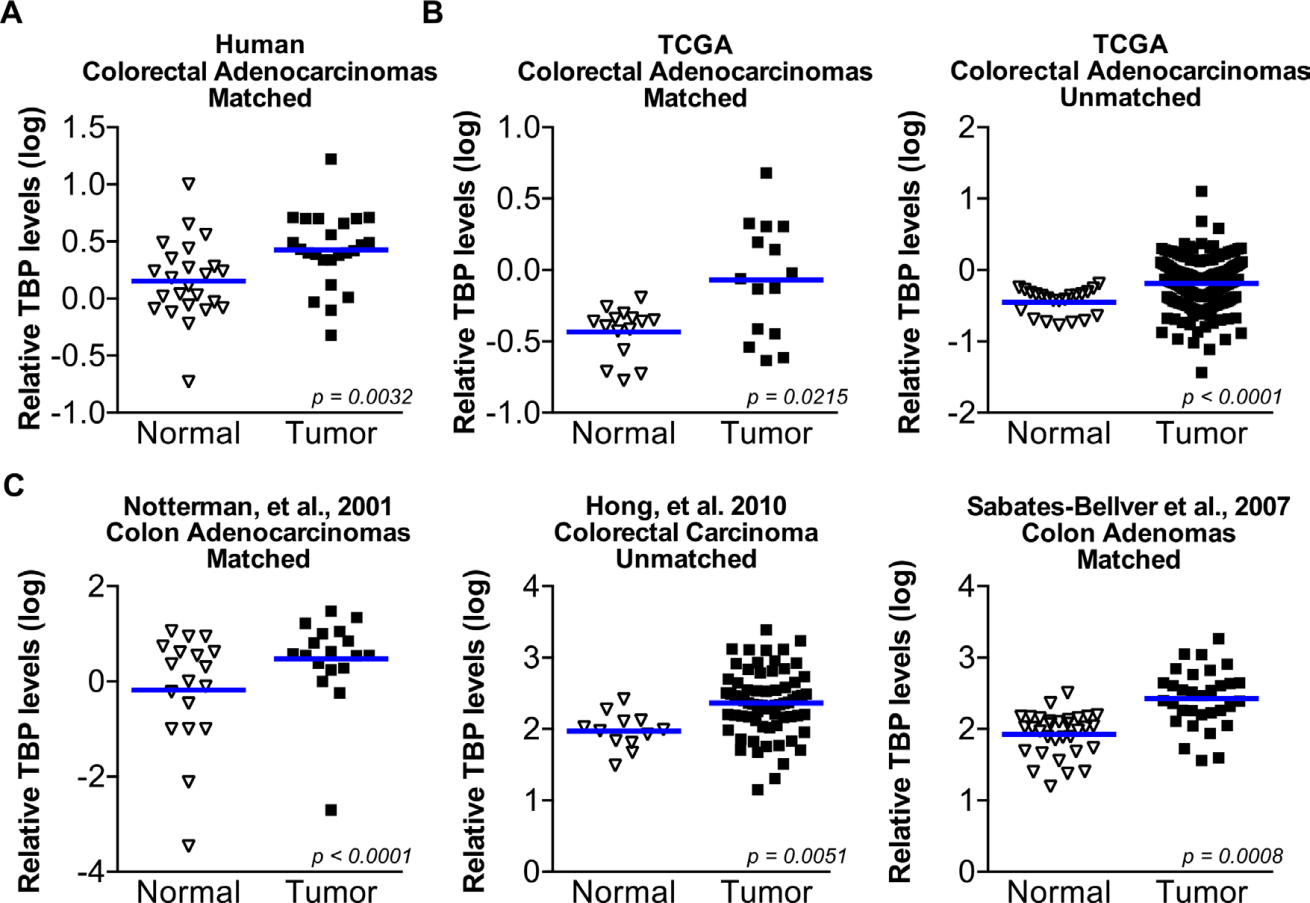
TBP is increased in a statistically significant number of human colorectal tumors compared to normal tissue (**A**) TBP transcripts determined by RT-qPCR in matched human colon tumor and normal colon epithelium from 24 patients. Cells were collected by LCM. Log values graphed. Line represents median TB*P* values. Median TB*P* values: normal (0.12) v. tumor (0.41), *n* = 24. (**B**) TBP expression data from microarrays from normal human colon tissue compared to colorectal tumors from publicly available TCGA data. *Right*. Matched comparison. Median TB*P* values: normal (−0.3615) v. tumor (−0.0595), *n* = 15. *Left*. Unmatched comparison. Median TB*P* values: normal (−0.3778, *n* = 22); tumor (−0.1515, *n* = 215). (**C**) TBP expression data from microarray in normal human colon compared to colorectal tumors from published sources. *Left*. Matched comparison. Median TB*P* values: normal (0.1498) v. tumor (0.5662), *n* = 18. *Middle*. Unmatched comparison. Median TB*P* values: normal (1.983, *n* = 12); tumor (2.359, *n* = 70). *Right*. Matched normal and colon adenomas. Median TB*P* values: normal (2.021) v. tumor (2.434), *n* = 32.

Our findings support the idea that enhanced TBP expression might be clinically important in human cancers. As our initial study showed that TBP expression was increased in several human colon cancer cell lines and tumors relative to normal, non-transformed cells [16], we further examined this in a larger patient population. Matched normal colon epithelium and neoplastic cells from biopsies of twenty-four patients were collected by laser-capture microdissection and subjected to qRT-PCR analysis. Using an adequate number of cases to provide sufficient statistical power, we found significant enrichment of TBP mRNA in tumor-derived epithelium compared to matched normal colon epithelium (Figure 7A). Comparing relative tumor TBP expression and the tumor stage or Duke's index did not reveal any association between increased tumor TBP expression and these factors (data not shown).

Oncomine was further used to identify datasets that measured changes in TBP expression between normal tissues and colorectal tumors. This *in silico* analysis revealed five datasets in which TBP was significantly increased in tumor compared to normal tissue. In both matched and unmatched samples from the TCGA database (Figure 7B) and additional three datasets from published sources (Figure 7C), relative TBP expression was significantly higher in colorectal tumors compared to normal tissue. Dataset analysis showed no significant association between TBP tumor expression and staging (data not shown). Furthermore, one of the datasets was comprised completely of adenomas, suggesting that enhanced TBP expression corresponds to an early event in colon cancer development.

### VEFGA expression is highly correlated with TBP expression in human colon tissue

**Figure 8:**
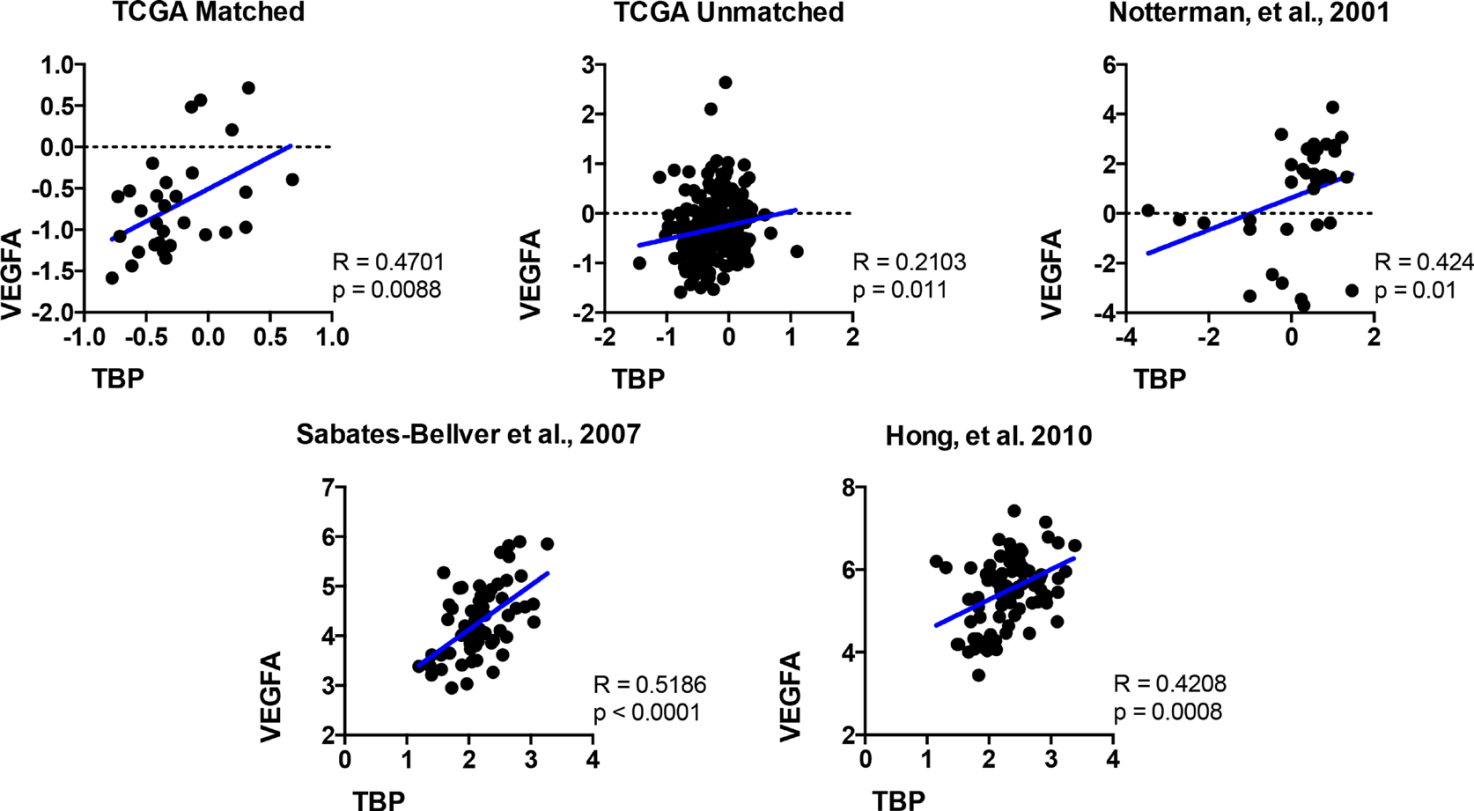
The relative expression of VEGFA and TBP are positively correlated in colon tissue TBP and VEGFA expression in normal human colon compared to colorectal tumors in public databases. Scatter plots for five datasets visualized positive correlations between relative TBP and VEGFA gene expression. TCGA matched, *n* = 30; TCGA unmatched, *n* = 237; Notterman *et al*., 2001, *n* = 36; Sabates-Beliver *et al*., 2007, *n* = 64; Hong *et al*., 2010, *n* = 82. Spearman correlation coefficient (R) and *p* values are shown.

Given our findings that TBP regulates VEGF expression, we compared relative TBP and VEGFA expression in normal tissue and colorectal tumors in the five publicly available datasets (Figure 8) as they were all shown to have increased TBP in tumors compared to the normal tissues (Figure 7). The relative TBP and VEGFA expression were strongly correlated (*R* > 0.4, *p* < 0.0001–0.0203) in four of these datasets and weakly, but still significantly, correlated in the remaining one (R = 0.2103, *p* = 0.011). Collectively, these results demonstrate a positive correlation between TBP and VEGFA in these human clinical biopsies, further supporting the idea that cellular TBP amounts play an important role in determining VEGFA production.

## DISCUSSION

Although alterations in TBP expression have been shown to differentially affect the transcription of specific RNA pol II-transcribed genes [3, 12–15], to date, no analyses have been carried out to comprehensively identify how TBP concentrations modulate overall changes in gene expression. As previous studies demonstrated that increases in cellular TBP concentrations can promote tumor formation in athymic mice [10, 16, 17], we sought to understand how this increase in TBP drives this process. Our analysis revealed that relatively small increases in TBP produce a limited number of gene expression changes. This suggests that in this cellular context the vast majority of mRNA encoded genes are insensitive to altered TBP expression and that the response is highly promoter dependent. Given that the microarray analysis revealed an enrichment of the angiogenesis pathway in the TBP-mediated transcriptome footprint, we specifically interrogated the potential role for TBP in driving VEGF expression. Using both human HT-29 and Rat1a cells we identified VEGF as a key TBP target that is sensitive to cellular TBP concentrations. Increased TBP expression induces VEGFA expression and enhances cell migration and tumor vascularization. While our analysis also revealed that there are other angiogenesis-enriched TBP-regulated genes that could conceivably contribute to the ability of TBP to regulate cell migration and tumorigenesis, changes in VEGF, alone, can drive tumor angiogenesis [19].

TBP's ability to induce VEGFA production and the importance of VEGFA in driving tumor growth prompted us to investigate whether TBP expression is elevated in human tumors relative to normal tissue. Our analysis of human colorectal cancer samples and datasets of matched and unmatched patient samples revealed that TBP is increased in a statistically significant number of tumors relative to normal epithelium. The finding that enhanced TBP expression is observed in a clinically significant population of human colorectal cancers supports the idea that TBP may function as an oncogene. Consistent with the ability of TBP to control VEGFA expression, we further find a positive correlation between relative TBP expression and VEGFA expression in colon tissues using several datasets. Recently, the expression of VEGFA has been identified as one of 15 genes that may serve as a predictor of recurrence risk and prognosis for colon cancer patients [36]. Given the strong correlation between VEGFA and TBP expression in colon cancer, TBP expression may represent a novel biomarker.

Given that TBP expression is statistically increased in at least one cohort of colorectal adenomas relative to normal colon, dysregulation of TBP expression may be an early event in tumor development. The formation of colon adenomas is strongly associated with the development of adenocarcinoma and a major risk factor for colon cancer. Previous work demonstrated that angiogenesis is induced early on in both human and animal models [27] and that limiting angiogenesis inhibits tumor growth and promotes tumor regression [20]. Increased TBP expression in adenomas may produce a constitutive increase in VEGFA expression that then primes these tissues for a more robust VEGFA induction under hypoxic conditions to generate an angiogenic switch.

TBP expression is upregulated by oncogenic signaling pathways that activate Ras and its downstream targets [1–3, 5, 11, 17]. Ras activation also induces VEGF expression and secretion [37, 38]. VEGF expression is positively correlated with the degree of Ras activation during mouse skin carcinogenesis and this is initiated early in premalignant papillomas [39]. Thus, Ras co-regulates VEGFA and TBP expression, consistent with the strong correlation between VEGFA and TBP expression in human colon tissue. Collectively, these results suggest that Ras signaling-induced increases in cellular TBP levels contribute to the observed Ras-mediated stimulation of VEGFA production early in oncogenesis. Since the oncogenic signaling pathways that regulate TBP expression are deregulated in many human tumor types [3–6, 11, 40] it is likely that enhanced TBP expression occurs in other human cancers as well.

Mechanisms that contribute to the induction of VEGFA expression that are influenced by the tumor microenvironment have been extensively studied. Transcriptional regulation of VEGFA plays a major role in controlling its expression and is stimulated by conditions including hypoxia, oxidative stress, ultraviolet radiation, and by hormones, growth factors, and cytokines [22, 41]. During hypoxia-dependent stimulation of VEGFA expression, the distal TSS alone is sufficient for induction and this is mediated by the recruitment of HIF-1, SP-1, and AP-1 transcription factors to specific sites upstream of this region [22, 41, 42]. In addition, Stat3, Egr-1, and AP-2 have all been shown to bind to the promoter and contribute to its activation both independent of, and in cooperation with, HIF-1 mediated induction.

In contrast to the understanding of activator-induced VEGFA expression, little is known about the mechanisms that confer constitutive activity of the VEGFA promoter. Many tumor cell lines constitutively express and secrete VEGFA independent of oxygen tension and the vast majority of human tumors have been shown to express VEGFA [18, 20]. Constitutive expression of VEGFA is dictated by the genetic composition of the tumor and this is considered a critical driver of tumor initiation [41]. Although the majority of analyses measured transcripts generated from the distal promoter, one study defined a novel proximal TSS and revealed that deletion of upstream sequences including the distal promoter still conferred transcription [25]. This proximal TSS is insensitive to hypoxia indicating that this promoter may provide a mechanism for ensuring constitutive VEGFA expression. Our results demonstrate an important role for this proximal promoter in driving VEGFA transcription and reveal that its activity is regulated by cellular TBP concentrations. Both our ChIP and published ChIP-seq datasets demonstrate that TBP is detected at both regions containing the distal and proximal VEGFA TSSs, although the relative occupancy at these sites varies depending on the cell type. Manipulating TBP expression in HT-29 cells predominately affects TBP binding to the proximal TSS, positively correlating with RNA pol II occupancy and transcription. This indicates that TBP controls expression of VEGFA via its ability to be recruited to the proximal TSS. The distal TSS contains an appropriately positioned TATA sequence and mutation of this site abolishes transcription [35, 43]. In contrast, the proximal TSS does not contain a discernable consensus TBP binding site. Our results indicate that this TATA-lacking proximal promoter is highly sensitive to altered TBP levels. This contrasts previous work that indicated that TATA-containing, but not TATA-lacking, promoters are responsive to alterations in cellular TBP amounts [12, 13]. Our results reveal that the sensitivity of RNA pol II-driven promoters to altered TBP levels is not simply due to the presence or absence or a TATA promoter and suggest that further analysis is needed to determine the basis for the different sensitivities of promoters to cellular TBP concentrations. In this particular case, usage of the VEGF proximal promoter could confer selective advantage for cells in that it produces a transcript that contains an IRES sequence that permits cap-independent translation under stress conditions [25]. This would allow the transcript to escape inhibitory translational regulation within the VEGFA 5′UTR. Together, our results support the idea of a mechanism by which cells may initially modulate VEGFA expression predominantly via the proximal TSS until the microenvironment creates signals that robustly induces VEGFA expression via the distal TSS.

Much is now known regarding the regulation of sequence-specific and chromatin modifying transcription factors that affect the recruitment of the basal transcription machinery. However, comparatively little is known about how the core promoter-interacting transcription factors, such as TBP, are regulated, and how this affects gene expression to control physiological processes. In fact many studies have assumed that, because of its central role in transcription, TBP is not regulated. While our results underscore the idea that small changes in TBP expression alter the tumorigenic property of cells, alterations in cellular TBP concentrations also play an important role in cellular differentiation. During the terminal differentiation of myocytes, hepatocytes, and adipocytes, cellular TBP concentrations become significantly downregulated [44–46]. In these cases changes in TBP protein levels are more pronounced relative to the decreases in TBP mRNA suggesting that it is regulated post-transcriptionally. Accordingly, further studies revealed that the ubiquitin-proteasome pathway downregulates TBP protein levels during the terminal differentiation of myotubes [47]. This is accomplished by the coordinate regulation of specific ubiquitination and deubiquitination enzyme activities. Collectively, these studies demonstrate that TBP expression is finely controlled in different cellular contexts, employing distinct regulatory mechanisms to execute cell type specification and to control the oncogenic capacity of cells.

## MATERIALS AND METHODS

### Tissues and cell lines

Tumorigenicity assays with athymic mice and Rat1a cells were described previously [16]. Matched human colon cancer and normal colon epithelium were obtained, laser-capture microdissected, prepared, and RNA analyzed as described previously [16]. The sample size of 24 was determined *a priori* to detect a significant ≥ 2-fold difference in TBP mRNA expression between matched human colon cancer and normal colon epithelium (α = 0.05, Power = 0.80).

Lentiviral vectors expressing inducible (hemagglutinin) HA-tagged hTBP (pFTREW-E2TBP), FUIPW-rtTA, non-silencing and TBP-shRNAmirs (GIPZ-ns-shRNA, GIPZ-shRNA1, GIPZ-TBP-shRNA2) were described previously [48]. Viral titers were determined using Lenti-X qRT-PCR Titration Kit (Clontech) and FACS analysis. HT-29 cells infected with FUIPW-rtTA and FTREW-E2TBP were puromycin selected (2 μg/ml) then infected with GIPZ-shRNA vectors and selected with Zeocin (100 μg/ml).

### Quantitative Real-Time RT-PCR

RNA preparation and qPCR analysis using the comparative Ct (2^−ΔΔCt^) method are described previously [8]. QPCR was performed using KAPA SYBR FAST Universal 2X qPCR Master Mix. TBP (rat and human) [10] and GAPDH primers [8] are previously published. Primer sets used are as follows: Rat-VEGFA: F_5′-TGCACTGGACCCTGGCTTTACTGC-3′; R_′-GCAG CCCGCACACCGCATT-3′; Human-VEGFA: F_5′-AGCC TTGCCTTGCTGCTCTA-3′, R_5′- GTGCTGGCCTTG GTGAGG-3′; TAF4: F_5′-ACAAGGATGACGACAG ATATGAGCAGG-3′, R_5′-CTGGATCTTCTTGTCTT- GACCGAGACT; R_5′- CTGGATCTTCTTGTCTTGA CCGAGACT; TAF15: F_5′- GGTGGCTATGGAGG CAAAATGGG-3′, R_5′- CGAGGAGCAGCAGGCAAA ACTC-3′.

### Immunohistochemical fluorescence

Frozen tissues in OCT were sectioned (5 μm), mounted on poly-L-lysine-coated and fixed in acetone. Fixed sections were blocked in 5% (v/v) goat serum in PBS then stained for von Willebrand factor overnight. Tissues were subsequently treated with FITC anti-rabbit secondary antibodies. Fluorescence was analyzed using Image J software (NIH). The scale for each image was determined and fluorescence threshold set > 10 μm^2^.

### Oncomine data

ONCOMINE datasets were selected using filters for “cancer versus normal analysis”, “colorectal cancer”, and “co-expression analysis”. Datasets were limited to those using commercially available microarrays and with > 5 normal tissue samples. Published datasets of human patient samples [49–51] and TCGA Network datasets (http://cancergenome.nih.gov) were identified. Median values from normal and tumor tissue types within each dataset were compared using Wilcoxon Signed Rank test (matched patient samples, normal v. tumor). Means of the ranked values were compared using Mann-Whitney test (unmatched normal v. tumor datasets). For each dataset, log transformed VEGFA and TB*P* values for each specimen were plotted on the *x* and *y*-axis, respectively, and Spearman correlation R and associated *p* values determined.

### Conditioned media, VEGF ELISA, and endothelial chemotaxis assay

VEGF levels in conditioned media from cultured cells post-plating (Rat1a, 48 h; HT29, 16 h) were determined using ELISA kits (rat or human VEGF, RayBiotech) and normalized to the number of cells plated. Human primary umbilical vein endothelial cells (HUVECs, ATCC) were cultured in EGM-2 media (Lonza) and were used in passages 3–4. Chemotaxis assays were performed as described previously [52].

### Immunoblot analysis and antibodies

Immunoblots were described previously [10] using HA-tag (Roche, clone 3F10), TBP (Santa Cruz, N-12), and b-actin (Millipore) antibodies. Bound primary antibodies were visualized using conjugated antibodies (biotinylated, HRP, or IRDye) and enhanced chemiluminescence reagents or IRDye conjugated secondary antibodies (Li-Cor) and visualized using infrared imaging (Li-Cor Odyssey, Lincoln, NE, USA).

### Encode data analysis

Density graphs of read count and signal enrichment for TBP was validated using SeqMonk and deepTools [53] and visualized using the Integrated Genomics Viewer [54] while window-averaged density graph figures were generated using tools of the Human Epigenome Browser, Washington University [55] Previously reported TBP ChIP-seq data [34] was obtained from ENCODE [53] Accession numbers: K562: ENCSR000EHA; HepG2: ENCSR000EEL; HeLa-S3: ENCSR000EDD; GM12878: ENCSR000DZZ.

### VEGF promoter reporter luciferase assays

Human VEGF promoter luciferase reporter is described previously [56]. A promoter fragment from −2278 to +371 was PCR amplified from −2278/+752 VEGF-luc and subcloned into pGL2 basic vector (Promega) at XhoI (5′) and HindIII (3′) to create −2278/+371D+633TSS VEGF-luc. Mutagenesis was performed to make −2278/+752 TATA mut VEGF-luc using QuikChange Lightning Mutagenesis Kit (Agilent) to change “T”->“G” at position 2 of a TATA-532 sequence, TTTAAAAG (−28 to −21) [35] to yield TGTAAAAG [43] HT-29 cells transfected with empty or TBP expression vector, pLTR-E2TBP, [16] or HT-29 inducible TBP expressing cells ± TBPshRNAs were transfected with hVEGF-luc and CMV-β-galactosidase plasmids using Lipofectamine 2000. Post-transfection (24 h), HT-29 inducible TBP cells were treated with doxycycline for 16 h. Lysates were prepared as described [1, 3].

### Chromatin immunoprecipitation (ChIP)

ChIP sample preparation from HT29 cells was described previously [57]. QPCR of chromatin fragments used KAPA SYBR FAST Universal 2X qPCR Master Mix. VEGFA promoter primer sets are relative to the distal transcription start site (−1038 bp of the translation start site) [25]: upstream control [−1694-F_5′-AGCAACATGTGCTGAGGATG-3′; −160 8-R_5′-GAATGGGAATGCAGCAATTT-3′]; [−349-F_ 5′-TTCCTAGCAAAGAGGGAACG-3′; −285-R_5′-AG GGAGCAGGAAAGTGAGGT-3′]; Distal transcription start site [−23-F_5′-AAGTCGGCTGGTAGCGGG-3′; +100 -R_ 5′-GCTGACCGGTCCACCTAACC-3′]; [+274-F_5′-ACTTCCCCAAATCACTGTGG-3′; +370-R_ 5′-GACCCCGTCTCTCTCTTCCT-3′]; Proximal transcription start site [+502-F_5′-CGGACAGACAGACAGACACCG-3′; +685 -R_ 5-CGAGAACAGCCCAGAAGTTG-3′]. Promoter occupancy is based on normalization to input and rabbit IgG control and target abundance is calculated based on primer efficiency for each target [1].

### Microarray analysis

We examined the impact of TBP overexpression in rat 1A cells via global gene expression profiling. RNA was reverse transcribed and microarray hybridization was performed using the Affymetrix platform. Transcriptome profile data was background-corrected and normalized using the rma function as implemented in the R *affy* package. Differences in gene expression of these samples were inferred utilizing the Bioconductor limma package [58] (*p* < 0.05) and imposing a fold change greater or equal than 1.25x or less or equal then 4/5x. For hierarchical clustering, gene expression of all TBP-regulated genes was first transformed to z-scores and then plotted using the R statistical system.

Gene Set Enrichment Analysis (GSEA) was performed using the GSEA software package [59] to assess the enrichment of known pathways. Genes were ranked by the fold change between the TBP overexpressed and the vector treated samples. Normalized Enrichment Score (NES) and adjusted *Q*-values were computed utilizing the GSEA method, based on 1000 random permutations of the ranked genes; significance was assessed for *Q* < 0.25. We performed GSEA using the Gene Ontology Biological Processes gene set collections GO as compiled by the Molecular Signatures Database compendium (http://www.broadinstitute.org/gsea/msigdb).

### Statistical analysis

Statistically significant differences were measured using *T*-tests, one-way analysis of variance (ANOVA) followed by Tukey's multiple comparisons test, or a two-way ANOVA followed by Sidak's multiple comparisons test where statistical differences were present. Reported “*n*” values represent independent experiments.

## SUPPLEMENTARY FILE




