# Supplementary material for: Elevated TATA-binding protein expression drives vascular endothelial growth factor expression in colon cancer
Source: Oncotarget. 2017 Mar 20;8(30):48832–45. doi: 10.18632/oncotarget.16384 (PMC5564728; doi:10.18632/oncotarget.16384)
Supplement: Supplementary file 1 [file oncotarget-08-48832-s001.pdf]

Elevated TATA-binding protein expression drives vascular endothelial growth factor expression in colon cancer

Supplementary Materials

Supplementary File 1: Gene Expression Signature. See Supplementary_File_1
